# The association between electronic health information usage and patient-centered communication: a cross sectional analysis from the Health Information National Trends Survey (HINTS)

**DOI:** 10.1186/s12913-023-10426-6

**Published:** 2023-12-12

**Authors:** Heidi Knowles, Thomas K. Swoboda, Devin Sandlin, Charles Huggins, Trevor Takami, Garrett Johnson, Hao Wang

**Affiliations:** 1 Department of Emergency Medicine, JPS Health Network, 1500 S. Main St, Fort Worth, TX 76104 USA; 2 Department of Emergency Medicine, The Valley Health System, Touro University Nevada School of Osteopathic Medicine, 657 N. Town Center Drive, Las Vegas, NV 89144 USA

**Keywords:** Health communication, Patient-centered, Personal health record, Internet

## Abstract

**Background:**

Patient-provider communication can be assessed by the patient-centered communication (PCC) score. With rapid development of electronic health (eHealth) information usage, we are uncertain of their role in PCC. Our study aims to determine the association between PCC and eHealth usage with the analysis of national representative survey data.

**Methods:**

This is a cross sectional analysis using the Health Information National Trends Survey 5 (HINTS 5) cycle 1 to cycle 4 data (2017–2020). Seven specific questions were used for PCC assessment, and eHealth usage was divided into two types (private-eHealth and public-eHealth usage). A multivariate logistic regression was performed to determine the association between PCC and eHealth usage after the adjustment of other social, demographic, and clinical variables.

**Results:**

Our study analyzed a total of 13,055 unweighted participants representing a weighted population of 791,877,728. Approximately 43% of individuals used private eHealth and 19% used public eHealth. The adjusted odds ratio (AOR) of private-eHealth usage associated with positive PCC was 1.17 (95% CI 1.02–1.35, p = 0.027). The AOR of public-eHealth usage associated with positive PCC was 0.84 (95% CI 0.71–0.99, p = 0.043).

**Conclusion:**

Our study found that eHealth usage association with PCC varies. Private-eHealth usage was positively associated with PCC, whereas public-eHealth usage was negatively associated with PCC.

## Introduction

### eHealth information (private and public eHealth)

With the rapid increase in internet use around the world, electronic health (eHealth) information has become another common healthcare resource available to individuals [1]. The most cited definition of eHealth was initially defined by Eysenbach in 2001 [1] as “health services and information delivered or enhanced through the internet and related technologies.” In recent years, after reviewing qualitative studies with key informants, a conceptual model for eHealth developed that included three prominent, overlapping eHealth domains: [2] (1) using digital technologies to monitor, track, and inform health, (2) using digital technologies to enable health communications between patients and healthcare providers, and (3) data enabling health. eHealth usage refers to the first two domains. Furthermore, eHealth information can also be divided into two categories: personal eHealth information restricted to only individuals and healthcare providers (e.g., individual patient portal or personal health records, usually referred to as protected health information, here after referred to as private-eHealth) and public eHealth information that individuals may share publicly (e.g., information shared in support groups, online forums, or social networking sites that might be open to the public with no access restriction, referred to as public-eHealth). Dealing with different formats of eHealth information may indicate patients having different preferences about their healthcare communication. Some might prefer directly communicating with healthcare providers, while others might seek online healthcare information to gain additional opinions [3].

### Patient-centered communication and eHealth

It is still uncertain whether seeking different eHealth information would affect patient-centered communication. Patient-centered communication (PCC) is a key component of patient-centered care and is directly related to healthcare quality [4, 5]. Reports from previous studies have shown indirect associations between PCC and eHealth usage [6–9]. Some studies have shown that individuals who had private-eHealth usage (e.g., patient portals) tended to promote better self-care behavior with better clinical outcomes [6, 7], a positive self-care behavior is positively associated with patient-centered communication [10]. Other studies suggested that individuals who had public-eHealth usage (e.g., online support group, apps to obtain online health-related information) could expand their health-related knowledge to help with making health-related decisions [8, 9], and patient-providers’ shared decision making is one of the key elements for patient-centered communication. However, other controversial findings can be found in the literature. Some studies showed no significant changes in terms of clinic outcomes among patients using patient portals versus ones who did not [11, 12]. Other studies showed online searching of health-related information might decrease patient’s perceptions of patient-centered communication [13]. Therefore, it is meaningful to further investigate how eHealth usage affects patient-provider rapport and subsequently influencing patient-centered communication utilizing large-scale data with diverse populations.

Previous studies have extended the use of PCC to further evaluate patient-provider rapport as a more efficient PCC is crucial for building up better patient-provider rapport [14, 15]. A PCC-scale can be calculated, with higher scores indicating better patient-centered communication [16]. Results from previous studies show that better PCC scores are linked to higher patient satisfaction, higher patient-provider trust, greater patient compliance, increased health-related self-efficacy, and improved clinical outcomes [17–20]. The PCC score can be used as an indicator for quality care. However, many factors could affect PCC scores including demographics, social determinant of health (SDOH), and individuals’ different approaches to healthcare information, etc. [3, 21]. For example, persons of older age tend to be associated with better PCC, and persons with lower incomes were associated with lower PCC [21]. However, reports on the different approaches to eHealth information (such as online information versus information from healthcare providers) and their effects on PCC are sparse.

### Clinical significance

To better understand eHealth affecting patient-provider rapport, determining the association between eHealth usage and patient-centered communication becomes an essential step. Factors such as providers’ attitude of dealing with their patients, providers’ body language, and the surrounding clinical environment could affect patients’ judgement of their communication leading to different perceptions of PCC [22, 23]. These factors are less perceived by patients during electronic communication, which could affect patient-provider communication effectively [24]. The formats of electronic communication can be virtual (e.g., telemedicine, chart messages), text message, or shared health information to an untargeted population (e.g., an online support group where individuals may receive feedback from a mix of healthcare and non-healthcare persons). The association between eHealth usage and PCC is uncertain. At present, relevant literature is mixed in their results and conclusions. Some studies report a positive association between eHealth usage (i.e., personal health record use) and PCC, [25, 26] while other studies reported a negative association between eHealth usage (i.e., telemedicine, searching publicly available health-related information) and PCC [13, 27].

### Objectives

There are differences between traditional face-to-face patient-provider encounters and eHealth communication [28]. Determining the associations between PCC and eHealth communication could identify factors that makes eHealth communication different from routine face-to-face communication. These findings could potentially serve as guidelines for implementation of improvements to electronic communication between patients and providers, with the goal of improving patient care outcomes. Taken together, our study aims to (1) investigate the differences of using two different eHealth usages (i.e., private- versus public-eHealth usage), and (2) determine the association between PCC and eHealth communication among individuals of different race/ethnicities.

## Methods

### Study designs and setting

This is a cross-sectional observational study. All data used in this study are de-identified data from one national representative survey and is publicly available at https://hints.cancer.gov/data/download-data.aspx. The regional institutional review board considered this research project as a non-human research study.

### Health Information National Trends Survey (HINTS)

HINTS is a nationally representative, cross-sectional survey of American adults that has been administered by the National Cancer Institute since 2003. Survey participants were randomly selected from adults aged 18 or older in the United States during the survey cycles. To determine the status of participants’ perception of PCC, their internet usage, their eHealth, and their use of usual sources of care, four HINTS data cycles were used (HINTS 5 cycle 1, 2, 3, and 4) in this study. HINTS 5 cycle 1 was conducted from January 25 through May 5, 2017, cycle 2 from January 26 through May 2, 2018, cycle 3 was conducted in 2 parts, the first one was conducted from January 22 to April 30, 2019, and another Web Pilot was conducted from January 29 through May 7, 2019. Cycle 4 was conducted from February 24 through June 15, 2020. The four cycles of HINTS data were appended using the merging tool of Stata data format (see website: https://hints.cancer.gov/data/data-merging-tool.aspx).

### The inclusion and exclusion criteria

The study population included all adults who participated in one of the HINTS 5 survey cycles. As this study focuses on the investigation of participants’ perception of PCC, participants whose PCC scores were missing or unable to be calculated (i.e., responded to less than 4 out of the 7 PCC questions) were excluded from this study. PCC scores indicate patients having communication with the healthcare providers, but they are unable to determine whether the communication was rendered via in-person or virtually. Our study assumes that patient-provider communication could occur in both formats. In addition, we did not exclude any participants whose PCC scores were calculated but reported having no usual care visits in the past 12 months. Patients could have visits which occurred more than 12 months ago, or they may have defined “provider visits” as in-person visits and did not count telemedical or virtual care as another type of healthcare visit.

### Outcome measures (patient-centered communication)

The primary study outcome was survey participants’ perceptions of PCC. In 2007, a group of seven questions was generated by the National Cancer Institute to comprehensively evaluate PCC. These questions correlate with the efficacy of building up patient-provider rapport and were included in the Health Information National Trends Survey (HINTS). These seven questions were: (1) How often did they give you the chance to ask all the health-related questions you had? (2) How often did they give the attention you needed to your feelings and emotions? (3) How often did they involve you in decisions about your health care as much as you wanted? (4) How often did they make sure you understood the things you needed to do to take care of your health? (5) How often did they explain things in a way you could understand? (6) How often did they spend enough time with you? and (7) How often did they help you deal with feelings of uncertainty about your health or health care?

In HINTS, a four-point Likert-scale is used to evaluate the levels of PCC for all 7 questions: Always, Usually, Sometimes, and Never. Scale scores are created by reverse-scoring all items (always = 4, usually = 3, sometimes = 2, and never = 1), summing all scores and taking the average. A minimum of four valid PCC item responses were chosen to generate a PCC score as previously reported [16]. The average PCC score ranges from 1.0 to 4.0. Since PCC scores are highly skewed data, the PCC score was further classified into two categories, positive perception was an average PCC score of 3.5 or greater and negative perception was an average PCC score of less than 3.5.

### Key Independent variables

#### Private eHealth usage

To evaluate private-eHealth usage, survey participants were asked, “How many times did you access your online medical record in the last 12 months?” This question determines which participants used private eHealth as these medical records are considered personal health information with restricted eHealth usage (e.g., restricted communication between patients and providers to protect privacy). Participants having at least one access to their personal online medical record were classified as positive private-eHealth users, whereas those who had not accessed their online medical record in the last 12 months were classified as negative users.

#### Public eHealth usage

To evaluate public-eHealth usage, survey participants were asked these two questions: “In the last 12 months, have you used the internet to share health information on social networking sites such as Facebook or Twitter?” and “In the last 12 months, have you used the internet to participate in an online forum or support group for people with a similar health or medical issue?” These health communications are open to both healthcare and non-healthcare persons and can become a publicly available online resource without restrictions. Positive public-eHealth users were identified if they answered “yes” to one or both questions while negative users were identified if they answered “no” to both questions.

#### Other key independent variables

Other variables include individual demographic, social, and health-related factors. Individual demographics included age (18–34, 35–49, 50–64, 65–74, and 75+), gender (male, female), race [Non-Hispanic White (NHW), Non-Hispanic Black (NHB), Hispanic/Latino, Non-Hispanic Asian (NHA), and other], and marital status (single, married, and other). Patient social factors included education level (less than high school, complete high school to some college level, and college and above), internet access (yes, no), and income level (<$20,000, $20,000–49,999, $50,000–99,999, and ≥$100,000). Patient health-related factors included insurance (yes, no), and usual source of care which was identified by the question “Not including psychiatrists and other mental health professionals, is there a particular doctor, nurse, or other health professional that you see most often?” Participants were considered as having a usual source of care if their answer was “yes.”.

### Data analysis

#### Missing data imputations

Participants were excluded who had either missing data from the outcome variable (PCC) or PCC scores were unable to be calculated. In terms of independent variables, deleting all participants who had missing data would generate significant participant selection bias. Therefore, all missing data from independent variables were imputed using multiple imputation by chained equations (MICE), an iterative form of stochastic imputation. All missing data was completed with estimated values to generate a complete data set. Such data generation was repeated 20 times. For each generation, a logistic regression for the binary variables (i.e., gender, race, insurance, having internet, regular source of care, private-eHealth, and public-eHealth), and multinomial logistic regression for unordered categorical variables (i.e., age, race, marital status, education level, and income level) was utilized. After 20 multiple imputed datasets were created, the specified estimation model on each dataset was executed and the final estimate was obtained with the combination of all the estimates across all the imputed datasets.

#### Statistical analysis

Participant general demographics were reported. Weighted percentages of each variable (internet use, routine source of care, private-eHealth access, public-eHealth access, and patient perception of PCC) were reported. These variables were reported and compared with different race/ethnicities (Non-Hispanic White (NHW), Non-Hispanic Black (NHB), Hispanic/Latino, Non-Hispanic Asian (NHA), and Other). In addition, multivariate logistic regression was performed to determine the association between individual perception of PCC and their eHealth activities. Adjusted odds ratios (AOR) with 95% of confidence interval (CI) were reported after the adjustment of other variables including participants demographic, social, and clinic-related factors. Due to analyzing the imputed data, the fractional amount of missing information (FMI) was calculated as higher FMI may indicate a problematic variable with a high proportion of missing information [29]. The number of data imputes should be equal to or greater than the largest FMI to ensure the accuracy of the estimates. In this study, all analyses including data merging, data imputation, and final analyses were performed by Stata 14.2 (College Station, TX).

### Reporting guideline

Strengthening of the reporting of observational studies in epidemiology (STROBE) reporting guidelines were followed in describing study methods and findings [30].

## Results

### Descriptive analysis

Four cycles of HINTS 5 data were merged with a total of 16,092 unweighted participants, including 3,285 participants from cycle 1, 3,504 from cycle 2, 5,438 from cycle 3, and 3,865 from cycle 4. 3,037 participants were excluded whose PCC scores cannot be calculated due to missing more than 3 PCC questions. A final 13,055 unweighted study participants representing 791,877,728 weighted individuals were placed into the analyses.

Table 1 shows the weighted percentages of all variables collected from this study before and after the multiple imputations. The missing data rates from all variables range from 0.17 to 12.59% with a median of 1.89%. Participants were female predominant (50.15%), Non-Hispanic White (NHW) individuals (61.97%), most participants had internet access (85.47%), insurance coverage (94.52%), and a usual source of care (72.96%). Less than half of the participants had private-eHealth (43.33%) and public-eHealth (18.85%) usages (see Table 1).

**Table 1:** A comparison of original and imputed variables used in the study

	Original (Wt%)	Imputed (Wt%)
Age		
18–34 35–49 50–64 65–74 75+ Missing/error	22.23% 24.96% 29.49% 12.16% 8.80% 2.35%	22.55% 25.35% 30.24% 12.71% 9.16%
Gender		
Male Female Missing/error	43.41% 50.15% 6.43%	46.05% 53.95%
Race/ethnicity		
Non-Hispanic White (NHW) Non-Hispanic Black (NHB) Hispanic/Latino Non-Hispanic Asian (NHA) Others Missing/error	61.97% 9.93% 13.29% 4.41% 3.10% 7.30%	65.96% 11.20% 14.77% 4.72% 3.35%
Education level		
Less than High School High school to Some college College and above Missing	6.91% 59.06% 32.14% 1.89%	7.15% 60.07% 32.77%
Marital status		
Single Married Others Missing	27.75% 55.28% 14.77% 2.20%	28.29% 56.19% 15.52%
Income ranges		
<$20,000 $20,000–49,999 $50,000–99,999 ≥$100,000+ Missing	16.79% 24.00% 30.57% 27.32% 1.33%	17.22% 24.38% 30.85% 27.55%
Insurance		
No Yes Missing/error	5.43% 93.91% 0.66%	5.48% 94.52%
Internet access		
No Yes Missing	14.49% 85.34% 0.17%	14.53% 85.47%
Usual source of care		
No Yes Missing	26.79% 72.48% 0.73%	27.04% 72.96%
Private-eHealth usage		
No Yes Missing	48.34% 39.07% 12.59%	56.67% 43.33%
Public-eHealth usage		
No Yes Missing	80.04% 18.70% 1.26%	81.15% 18.85%

Note: Table-1 illustrates the weighted percentages of variables before and after the imputation. The missing/error information of variables were all replaced after the imputation

### Main results

Internet access, usual source of care, eHealth activities, and PCC were compared among participants with different race/ethnicities. Table 2 shows Hispanic/Latino participants tended to have lower usual source of care (55.98%, p < 0.05 in comparison to NHW, NHB, and other race/ethnicities) and lower private-eHealth usage (33.56%, p < 0.05 in comparison to NHW and NHA). People who identified as Non-Hispanic Black (NHB) tended to have lower internet access (76.63%, p < 0.05 in comparison to NHW and NHA). Though NHW participants had a lower public-eHealth usage, no statistically significant differences were found (18.24%, p > 0.05 when compare with other 4 groups of race/ethnicity). NHA had the lowest perception of PCC (37.94%, p < 0.05) when compared to all other groups.

**Table 2:** PCC and eHealth usage comparisons among participants of different race/ethnicities

	NHW	NHB	Hispanic/Latino	NHA	Others
Internet access --- yes (Wt%)	88.18	76.63	78.75	86.33	89.74
Usual sources of care --- yes (Wt%)	78.74	67.78	55.98	61.38	66.76
Private-eHealth user --- yes (Wt%)	46.34	35.39	33.56	49.18	41.30
Public-eHealth user --- yes (Wt%)	18.24	20.83	19.31	19.22	21.86
Individual perception of positive PCC --- (Wt%)	55.13	56.52	50.65	37.94	51.40

*Note: Internet: p < 0.05 when NHB compared with NHW, NHA, and others; Usual source of care: p < 0.05 when Hispanic/Latino compared with NHW, NHB, and others; Private-eHealth: p < 0.05 when Hispanic/Latino were compared with NHW, and NHA; Public-eHealth: p > 0.05 when NHW compared with all other four groups; ideal PCC: p < 0.05 when NHA compared with all other groups*

A multivariate logistic regression (Table 3) was performed to determine the association between PCC and eHealth usage after adjusting for all other factors including demographics (age, gender, race/ethnicity, and marital status), social (internet access, income level, and education level), and clinical information (insurance coverage, and usual source of care). The adjusted odds ratio (AOR) of public-eHealth usage associated with PCC was 0.84 (95% CI 0.71–0.99, p = 0.043). The AOR of private-eHealth usage associated with PCC was 1.17 (95% CI 1.02–1.35, p = 0.027). In addition, two factors were associated with positive PCC including usual source of care (AOR = 1.36, 95%CI 1.15–1.59, p < 0.001) and higher income (≥$100,000+, AOR = 1.48, 95%CI 1.17–1.88, p = 0.001). Two factors were associated with negative PCC including having internet access (AOR = 0.79, 95% CI 0.65–0.96, p = 0.019) and being a NHA (AOR = 0.55, 95% CI 0.40–0.75, p < 0.001). The largest FMI was 12%, which indicates that at least 12 randomly imputed datasets are required for the accuracy of missing data imputation. Twenty repeated datasets were utilized in this study indicating that the imputed datasets were adequate.

**Table 3:** Association between PCC and eHealth usage

	Adjusted Odds Ratio with 95% CI	P value
Public-eHealth usage		
No Yes	Reference 0.84 [0.71, 0.99]	0.043
Private-eHealth usage		
No Yes	Reference 1.17 [1.02, 1.35]	0.027
Usual source of Care		
No Yes	Reference 1.36 [1.15, 1.59]	< 0.001
Internet access		
No Yes	Reference 0.79 [0.65, 0.96]	0.019
Insurance coverage		
No Yes	Reference 1.06 [0.75, 1.50]	0.750
Age		
18–34 35–49 50–64 65–74 75+	Reference 0.89 [0.71, 1.11] 0.92 [0.75, 1.13] 1.09 [0.88, 1.36] 0.87 [0.67, 1.12]	0.312 0.418 0.434 0.271
Gender		
Male Female	Reference 1.10 [0.96, 1.25]	0.158
Race/ethnicity		
NHW NHB Hispanic/Latino NHA Others	Reference 1.17 [0.95, 1.44] 0.94 [0.77, 1.14] 0.55 [0.40, 0.75] 0.93 [0.65, 1.34]	0.144 0.502 < 0.001 0.700
Marital status		
Single Married Others	Reference 1.03 [0.87, 1.23] 1.21 [0.99, 1.47]	0.710 0.058
Education level		
Less than High school High school to some college College and above	Reference 1.12 [0.86, 1.47] 0.95 [0.71, 1.27]	0.392 0.707
Income level		
<$20,000 $20,000–49,999 $50,000–99,999 ≥$100,000+	Reference 1.18 [0.96, 1.44] 1.13 [0.90, 1.40] 1.48 [1.17, 1.88]	0.115 0.288 0.001

*Note: A multivariate logistic regression using imputed data with replicate weights was performed to determine the association between PCC and all variables. AOR with 95% CI and p value were reported in Table-3*

We further performed the public versus private eHealth usage interaction analysis. Individuals were divided into 4 groups. Using Individuals with public but no private eHealth usage as the reference, the AOR of individuals with both private and public eHealth usage was 1.24 (95% CI 0.98–1.56, p = 0.071), AOR of individuals with neither private nor public eHealth usage was 1.30 (95% CI 1.04–1.62, p = 0.020). More importantly, AOR of individuals with private but no public eHealth usage had over 1.5-time higher odds to be associated with higher PCC scores (AOR = 1.58, 95% CI 1.27–1.98, p < 0.001), indicating the synergistic effect of different eHealth usage.

## Limitation

There are areas in which this study is limited. First, our study was a retrospective observational study, where missing data or data errors are unavoidable. Second, though our study combined 4 years of HINTS 5 data, we can only determine the recent trend of PCC and eHealth changes (e.g., from 2017 to 2020). Our methods could only determine the association between PCC and eHealth use without knowing any potential causative effect. Although all common independent variables for such association analysis have been included, other potential variables could exist that affect individual perception of patient-centered communication and act as a significant confounder to this analysis. Third, we did not exclude participants who had PCC scores but did not have usual care in the past 12 months. Though we interpreted them as those who may have had a visit more than 12 months ago or have had virtual care visits, there were no direct questions to participants that would validate our assumption. Additionally, no questions focused on telehealth visits, therefore, we determined PCC associated with both in-person and virtual patient-provider communication. Finally, since two variables had “other” categories (i.e., race/ethnicity and marital status), due to their relatively small sample sizes, our study combined the data and classified it under the “other” category, which might cause misclassification bias. A future study focusing on a comprehensive analysis of factors affecting PCC (such as in-person versus virtual health visits, patient-provider trust, confidence in getting online health-related information, more detailed sociodemographic variables, etc.) and eHealth communication is warranted.

## Discussion

### Study main findings

As usage of the internet has increased, eHealth usage has become a more important means of communication in the field of healthcare [31, 32]. Our study investigates the association between patient-centered communication (PCC) and eHealth use and found that that association varies. Using protected/restricted eHealth information (referred to as private-eHealth) was found to have a positive PCC association while using unprotected/unrestricted eHealth information (referred to as public-eHealth) had a negative association with PCC. This may indicate that patient-provider direct communication is important. Private eHealth use focused on the patient portal use in HINTS, and patient portal use is associated with positive PCC in the literature [25]. On the contrary, public eHealth with no restricted information and no focused communication target, often acted as an adjunct means to obtain needed health information. However, the quality of online health information varies, inconsistency might be found between online eHealth information and health information provided from healthcare providers, this could, potentially affect the patient-provider trust, thus influencing PCC [13]. These findings suggest that providers should pay more attention to patients’ preferences about approaching eHealth information. With the current trend of easily getting online health information, providers might need to discuss the value of health information released in public. Providers might need to help patients screen the content of their eHealth information and identify the value of using it with online resources. Given the complexity of eHealth usage within different formats, specific quality assessment tools targeting different eHealth formats might need to be developed. Our study’s results could serve as a foundation towards the development of healthcare provider education and appropriate quality assessment tools for future eHealth usage.

### PCC and eHealth findings

Similar findings have been reported in past studies. Individuals’ PCC has been positively associated with personal health record use (e.g., patient portal) [25, 33]. Patients who used a patient portal more frequently tended to have better self-management behavior especially among ones with multimorbidity [19, 34]. Great patient-centered communication could also promote patient self-care behavior [10]. Taken together, it seems that an individual patient’s level of private-eHealth usage (e.g., patient portal use, or electronic communication between patients and healthcare providers, etc.) might improve patient-centered communication.

Public-eHealth usage can target the general population without focusing on any specific individual. Previous studies showed that decreased PCC may be related to increased online viewing of health-related topics, [13, 35] possibly because of poor patient-centered communication. Since patients might not fully understand their health conditions, or healthcare providers might not explain information clearly, individuals may search additional health-related information [13]. Another explanation may be a lack of trust in healthcare providers, leading patients to seek out external resources to have second opinions or help make their medical decisions [36].

Electronic health communication differs from traditional health communication because it largely relies on digital technology (such as digital devices and the internet) with different communication formats (such as virtual video, text message, or online resources). This communication can be one-to-one (e.g., e-communication with certain healthcare providers) or one-to-multiple persons (e.g., forum or support group discussion). The setting of eHealth communication can vary over different time frames or in different settings (e.g., texting message while doing other tasks simultaneously). Health literacy also differs from eHealth literacy thus making the assessment of eHealth communication different than face-to-face communication [37].

### Other factors influencing PCC

In addition, having usual sources of care was associated with positive PCC (Table 3) which might be caused by regular healthcare providers encouraging patients to use their patient portal [38]. Our study also found disparities in terms of perceptions of communication. NHWs tended to have lower public- and private-eHealth usages than the NHA group; but their perception of PCC was higher than NHA who tended to have both higher levels of public- and private-eHealth activities. Though similar findings have been reported in the literature, their mechanism(s) are still unclear [39, 40]. This might be attributed to NHA having relatively higher levels of education than other race/ethnicities as individuals with higher levels of education have been associated with relatively lower perception of PCC [41]. In addition, other factors might also affect NHA perception of PCC when compared with NHW including lack of understanding Asians’ cultural background and providers’ cognitive biases. When providers taking care of NHA patients, they might subconsciously consider NHAs’ having received higher level of education, thus may give less explanation of the treatment regimen and follow-up details, leading to the poor perception of PCC among such individuals [42–44]. Our findings are consistent with previous reports of other factors associated with PCC (e.g., higher income, usual source of care), indicating these common risks should be analyzed together to avoid the potential confounders [21, 41].

Our study imputed independent variables by using MICE method. A significant amount of missing data would decrease the data accuracy if all were excluded and cause significant data selection bias. Multiple imputation using chained equations (MICE) produces unbiased coefficient estimates [45]. Our study created 20 imputed datasets to enhance the accuracy. The association analysis on different types of eHealth usage was performed simultaneously with PCC after the adjustment of other potential confounders. Meanwhile, private versus public eHealth interaction analysis showed that less public eHealth usage in combination with more private eHealth usage might significantly improve PCC. However, such findings may require further validations. Our future study will further investigate the differences between traditional face-to-face and eHealth communication with the purpose of deriving suitable communication quality assessment tools for eHealth communication, identify potential interventions to improve private eHealth usage to further improve PCC, and determine the mechanism(s) of public eHealth use in related to the poor PCC among different racial and ethnic populations.

## Conclusion

Our study found that the association of eHealth usage with PCC varies based on type. Private-eHealth usage was positively associated with PCC, whereas public-eHealth usage was negatively associated with PCC.

## Data Availability

The datasets generated and/or analyzed during the current study are available at https://hints.cancer.gov/data/download-data.aspx.

## References

[1] Eysenbach G (2001). What is e-health?. J Med Internet Res.

[2] Shaw T, McGregor D, Brunner M, Keep M, Janssen A, Barnet S (2017). What is eHealth (6)? Development of a conceptual model for eHealth: qualitative study with Key informants. J Med Internet Res.

[3] Hou J, Shim M. The role of provider-patient communication and trust in online sources in Internet use for health-related activities. J Health Commun. 2010;15 Suppl 3:186 – 99. doi: 10.1080/10810730.2010.522691. PubMed PMID: 21154093. 10.1080/10810730.2010.522691 21154093

[4] Newell S, Jordan Z (2015). The patient experience of patient-centered communication with nurses in the hospital setting: a qualitative systematic review protocol. JBI Database System Rev Implement Rep.

[5] Blanch-Hartigan D, Chawla N, Beckjord EI, Forsythe LP, de Moor JS, Hesse BW (2015). Cancer survivors’ receipt of treatment summaries and implications for patient-centered communication and quality of care. Patient Educ Couns.

[6] Shimada SL, Allison JJ, Rosen AK, Feng H, Houston TK (2016). Sustained use of patient portal features and improvements in Diabetes physiological measures. J Med Internet Res.

[7] Morrow D, Hasegawa-Johnson M, Huang T, Schuh W, Azevedo RFL, Gu K (2017). A multidisciplinary approach to designing and evaluating Electronic Medical Record portal messages that support patient self-care. J Biomed Inform.

[8] Mackintosh N, Agarwal S, Adcock K, Armstrong N, Briley A, Patterson M (2020). Online resources and apps to aid self-diagnosis and help seeking in the perinatal period: a descriptive survey of women’s experiences. Midwifery.

[9] Laukka E, Rantakokko P, Suhonen M (2019). Consumer-led health-related online sources and their impact on consumers: an integrative review of the literature. Health Inf J.

[10] Berg CA, Campbell MS, Kent de Grey RG, Butner JE, Murray M, Wiebe DJ (2022). Parental relationships, patient-centered communication with Healthcare Providers, and Diabetes Management Across Emerging Adulthood. J Pediatr Psychol.

[11] Dumitrascu AG, Burton MC, Dawson NL, Thomas CS, Nordan LM, Greig HE (2018). Patient portal use and hospital outcomes. J Am Med Inform Assoc.

[12] Tome J, Ahmed S, Fagerlin A, Powell C, Mourao M, Chen E et al. Patient Electronic Health Record Portal Use and Patient-Centered Outcomes in CKD. Kidney Med. 2021;3(2):231 – 40 e1. Epub 20210209. doi: 10.1016/j.xkme.2020.11.014. PubMed PMID: 33851118; PubMed Central PMCID: PMCPMC8039427. 10.1016/j.xkme.2020.11.014 PMC8039427 33851118

[13] Langford A, Loeb S (2019). Perceived patient-provider communication quality and sociodemographic factors Associated with watching Health-related videos on YouTube: a cross-sectional analysis. J Med Internet Res.

[14] Jiang S (2020). The relationship between Face-to-Face and Online patient-provider communication: examining the Moderating Roles of Patient Trust and patient satisfaction. Health Commun.

[15] Dang BN, Westbrook RA, Njue SM, Giordano TP (2017). Building trust and rapport early in the new doctor-patient relationship: a longitudinal qualitative study. BMC Med Educ.

[16] Moser RP, Trivedi N, Murray A, Jensen RE, Willis G, Blake KD (2022). Patient-centered communication (PCC) scale: psychometric analysis and validation of a health survey measure. PLoS ONE.

[17] Finefrock D, Patel S, Zodda D, Nyirenda T, Nierenberg R, Feldman J (2018). Patient-centered communication behaviors that correlate with higher patient satisfaction scores. J Patient Exp.

[18] Croom A, Wiebe DJ, Berg CA, Lindsay R, Donaldson D, Foster C (2011). Adolescent and parent perceptions of patient-centered communication while managing type 1 Diabetes. J Pediatr Psychol.

[19] Finney Rutten LJ, Hesse BW, St Sauver JL, Wilson P, Chawla N, Hartigan DB (2016). Health Self-Efficacy among populations with multiple chronic conditions: the value of patient-centered communication. Adv Ther.

[20] Benner JS, Erhardt L, Flammer M, Moller RA, Rajicic N, Changela K (2008). A novel programme to evaluate and communicate 10-year risk of CHD reduces predicted risk and improves patients’ modifiable risk factor profile. Int J Clin Pract.

[21] Trivedi N, Moser RP, Breslau ES, Chou WS (2021). Predictors of patient-centered communication among U.S. adults: analysis of the 2017–2018 Health Information National trends Survey (HINTS). J Health Commun.

[22] Ghabowen IK, Bhandari N (2021). Concordance and patient-centered care in Medicaid enrollees’ Care Experience with providers. J Patient Exp.

[23] de Rezende Rde C, de Oliveira RM, de Araujo ST, Guimaraes TC, do, Espirito Santo FH, Porto IS. Body language in health care: a contribution to nursing communication. Rev Bras Enferm. 2015;68(3):430-6, 90 – 6. 10.1590/0034-7167.2015680316i. PubMed PMID: 26312521. 10.1590/0034-7167.2015680316i 26312521

[24] Paradisi P, Raglianti M, Sebastiani L (2021). Online Communication and Body Language. Front Behav Neurosci.

[25] Zaidi M, Amante DJ, Anderson E, Ito Fukunaga M, Faro JM, Frisard C (2022). Association between Patient Portal Use and Perceived patient-centered communication among adults with Cancer: cross-sectional survey study. JMIR Cancer.

[26] Lear R, Freise L, Kybert M, Darzi A, Neves AL, Mayer EK (2022). Perceptions of quality of Care among users of a web-based patient Portal: cross-sectional survey analysis. J Med Internet Res.

[27] Paige SR, Bunnell BE, Bylund CL (2022). Disparities in patient-centered communication via Telemedicine. Telemed J E Health.

[28] Liu X, Sawada Y, Takizawa T, Sato H, Sato M, Sakamoto H (2007). Doctor-patient communication: a comparison between telemedicine consultation and face-to-face consultation. Intern Med.

[29] Madley-Dowd P, Hughes R, Tilling K, Heron J (2019). The proportion of missing data should not be used to guide decisions on multiple imputation. J Clin Epidemiol.

[30] von Elm E, Altman DG, Egger M, Pocock SJ, Gotzsche PC, Vandenbroucke JP (2007). Strengthening the reporting of Observational studies in Epidemiology (STROBE) statement: guidelines for reporting observational studies. BMJ.

[31] Ossebaard HC, Van Gemert-Pijnen L (2016). eHealth and quality in health care: implementation time. Int J Qual Health Care.

[32] Tossaint-Schoenmakers R, Versluis A, Chavannes N, Talboom-Kamp E, Kasteleyn M (2021). The Challenge of Integrating eHealth Into Health Care: systematic literature review of the Donabedian Model of structure, process, and Outcome. J Med Internet Res.

[33] Totzkay D, Silk KJ, Sheff SE (2017). The Effect of Electronic Health Record Use and patient-centered communication on Cancer Screening Behavior: An Analysis of the Health Information National Trends Survey. J Health Commun.

[34] Wang H, Ho AF, Wiener RC, Sambamoorthi U. The Association of Mobile Health Applications with Self-Management Behaviors among Adults with Chronic Conditions in the United States. Int J Environ Res Public Health. 2021;18(19). Epub 20210930. 10.3390/ijerph181910351. PubMed PMID: 34639651; PubMed Central PMCID: PMCPMC8507726. 10.3390/ijerph181910351 PMC8507726 34639651

[35] Senft N, Everson J (2018). eHealth Engagement as a response to negative Healthcare Experiences: cross-sectional survey analysis. J Med Internet Res.

[36] Li N, Orrange S, Kravitz RL, Bell RA (2014). Reasons for and predictors of patients’ online health information seeking following a medical appointment. Fam Pract.

[37] Monkman H, Kushniruk AW, Barnett J, Borycki EM, Greiner LE, Sheets D. Are health literacy and eHealth literacy the same or different? Stud Health Technol Inform. 2017;245:178 – 82. PubMed PMID: 29295077. 29295077

[38] Horhammer I, Kujala S, Hilama P, Heponiemi T (2021). Building Primary Health Care Personnel’s support for a patient Portal while alleviating ehealth-related stress: Survey Study. J Med Internet Res.

[39] Arora NK, Reeve BB, Hays RD, Clauser SB, Oakley-Girvan I (2011). Assessment of quality of cancer-related follow-up care from the cancer survivor’s perspective. J Clin Oncol.

[40] Acoba JD, Yin C, Meno M, Abe J, Pagano I, Tamashiro S (2022). Racial disparities in patient-provider communication during Telehealth visits Versus face-to-face visits among Asian and native hawaiian and other Pacific Islander patients with Cancer: cross-sectional analysis. JMIR Cancer.

[41] Blanch-Hartigan D, Chawla N, Moser RP, Finney Rutten LJ, Hesse BW, Arora NK (2016). Trends in cancer survivors’ experience of patient-centered communication: results from the Health Information National trends Survey (HINTS). J Cancer Surviv.

[42] Ngo-Metzger Q, Legedza AT, Phillips RS (2004). Asian americans’ reports of their health care experiences. Results of a national survey. J Gen Intern Med.

[43] Bodenhausen GV (2005). The role of stereotypes in decision-making processes. Med Decis Making.

[44] van Ryn M. Research on the provider contribution to race/ethnicity disparities in medical care. Med Care. 2002;40(1 Suppl). 10.1097/00005650-200201001-00015. PubMed PMID: 11789627. :I140-51. 10.1097/00005650-200201001-00015 11789627

[45] Azur MJ, Stuart EA, Frangakis C, Leaf PJ (2011). Multiple imputation by chained equations: what is it and how does it work?. Int J Methods Psychiatr Res.

